# DEBT AMONG OLDER ADULTS: DATA FROM AN EXPLORING STUDY IN SOUTH BRAZIL

**DOI:** 10.1093/geroni/igz038.458

**Published:** 2019-11-08

**Authors:** Johannes Doll, Francieli Conte, Karen Lima

**Affiliations:** School of Education, Universidade Federal do Rio Grande do Sul, Porto Alegre, Brazil

## Abstract

In recent years, Brazil has experienced significant increase in indebtedness rates amongst older adults. This is partly due to prominent forms of credit such as credit cards and payroll deductible credit, a type of credit that accounts for up to 35% of pensions. In addition, indebtedness is aggravated by high interest rates which are charged in Brazil. Exploratory study on the propensity for indebtedness amongst older people in Rio Grande do Sul, Southern Brazil (n = 406; age 46-97 years old; average 68,7 years old) aims to analyze reasons and factors which interfere in the financial-related issue. In general, indebtedness is a complex process that is often triggered by critical life events such as illness, death, separation, accident and unemployment. And, it is mediated by a number of behavioral and sociodemographic factors. The present work aims to analyze the relation between financial problems, income, schooling, age, materialism, consuming habits as well as altruism in two different social groups: a middle class (SESC) and a lower middle-class (FASC). Concerning theoretical reference, data discussion is based on Ronald Inglehart’s theory on Materialism and Postmaterialism. Data analysis has shown differences in factors and relations among the two social groups. Consuming habits and altruism have a significant correlation with financial problems in both groups. However, income, education and materialism has shown a significant correlation only in the lower middle-class group. In the middle-class group, no relation between income, education, materialism and financial problems has been found.

